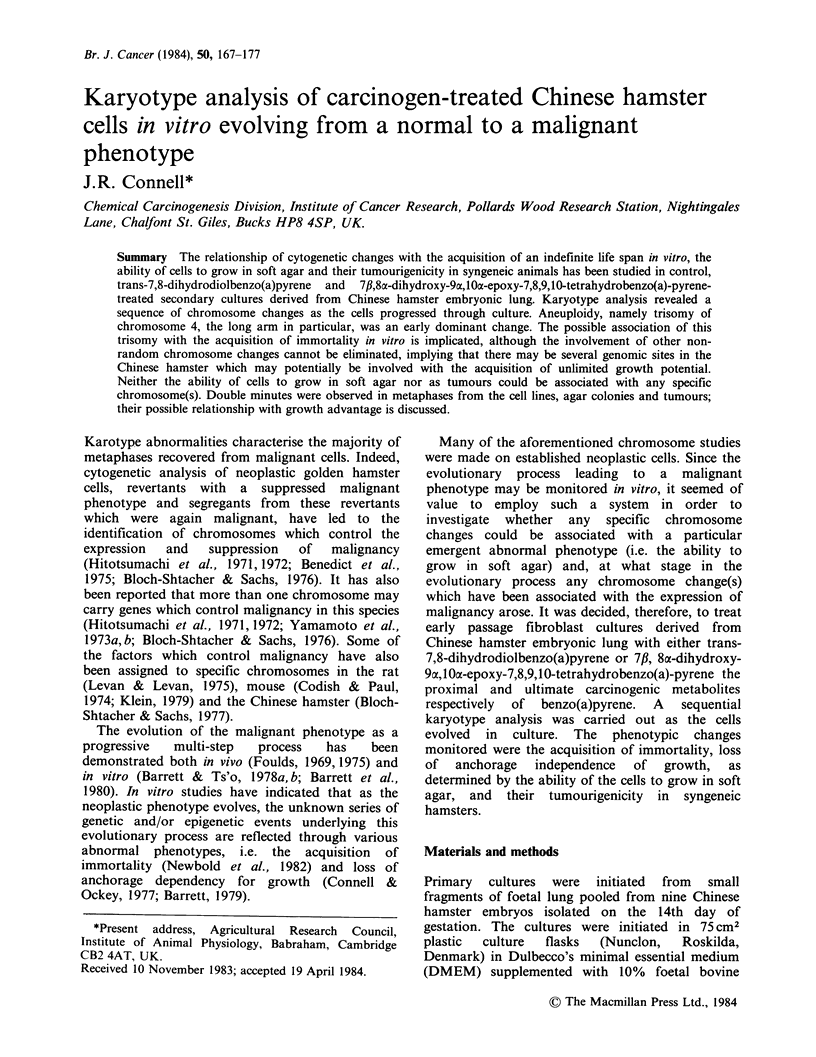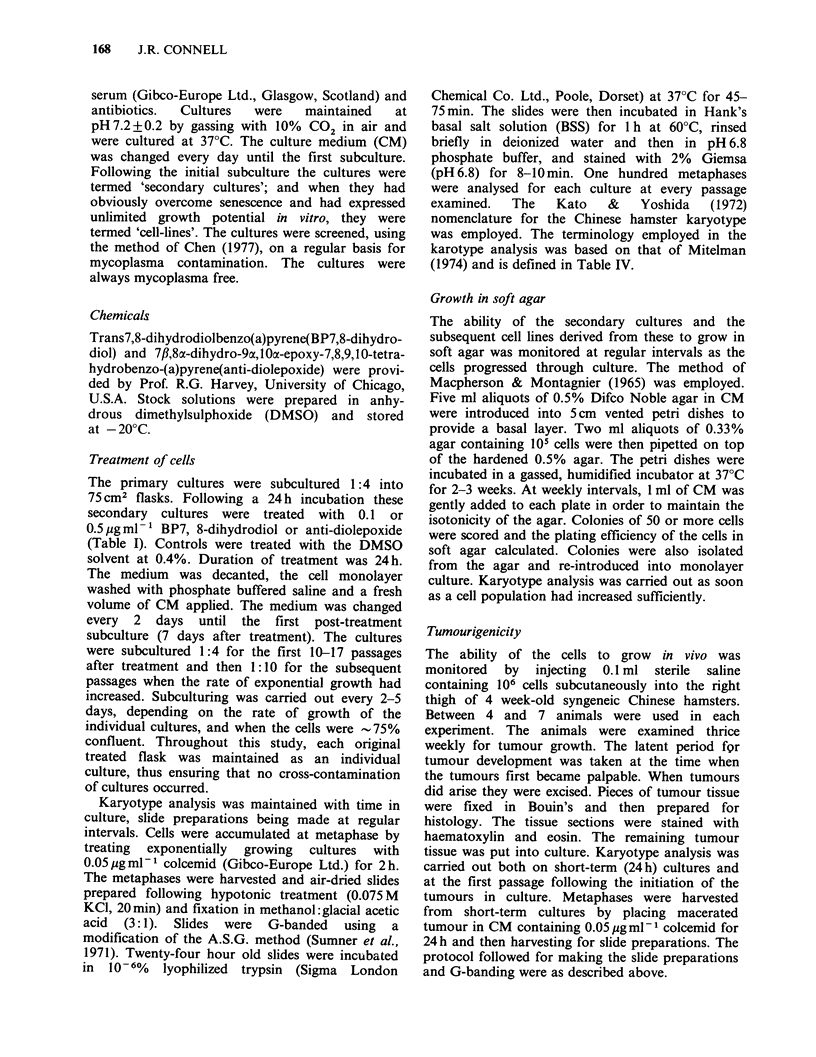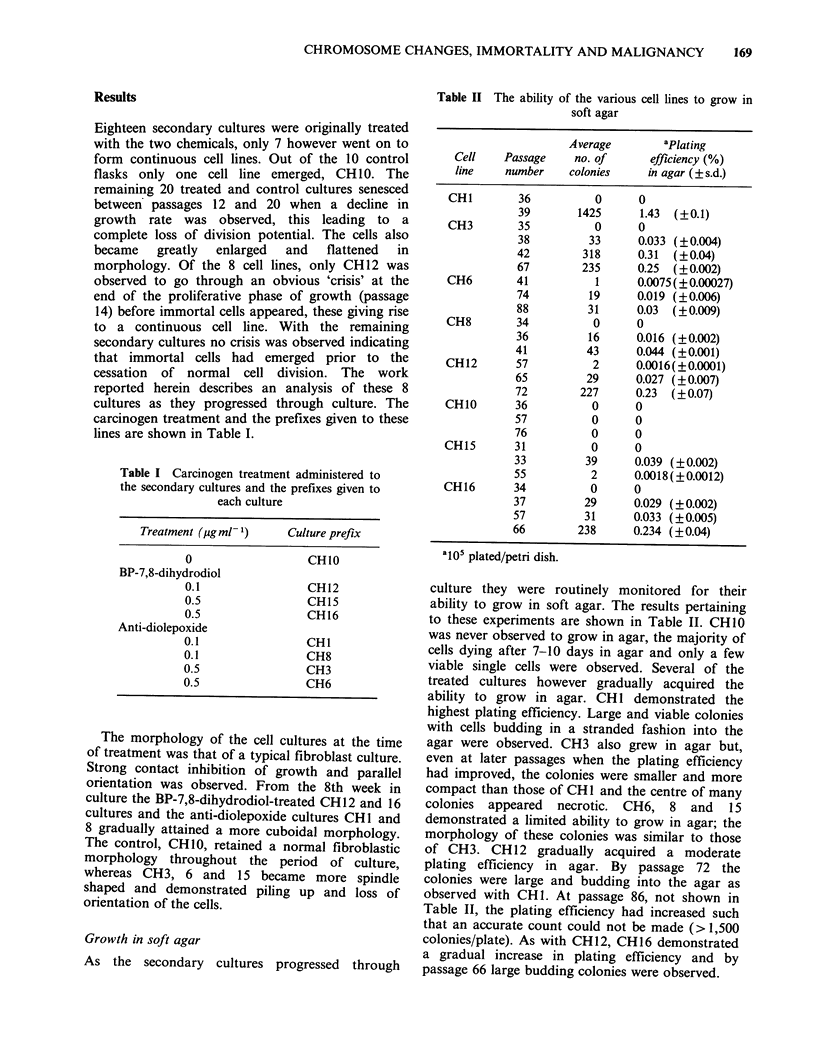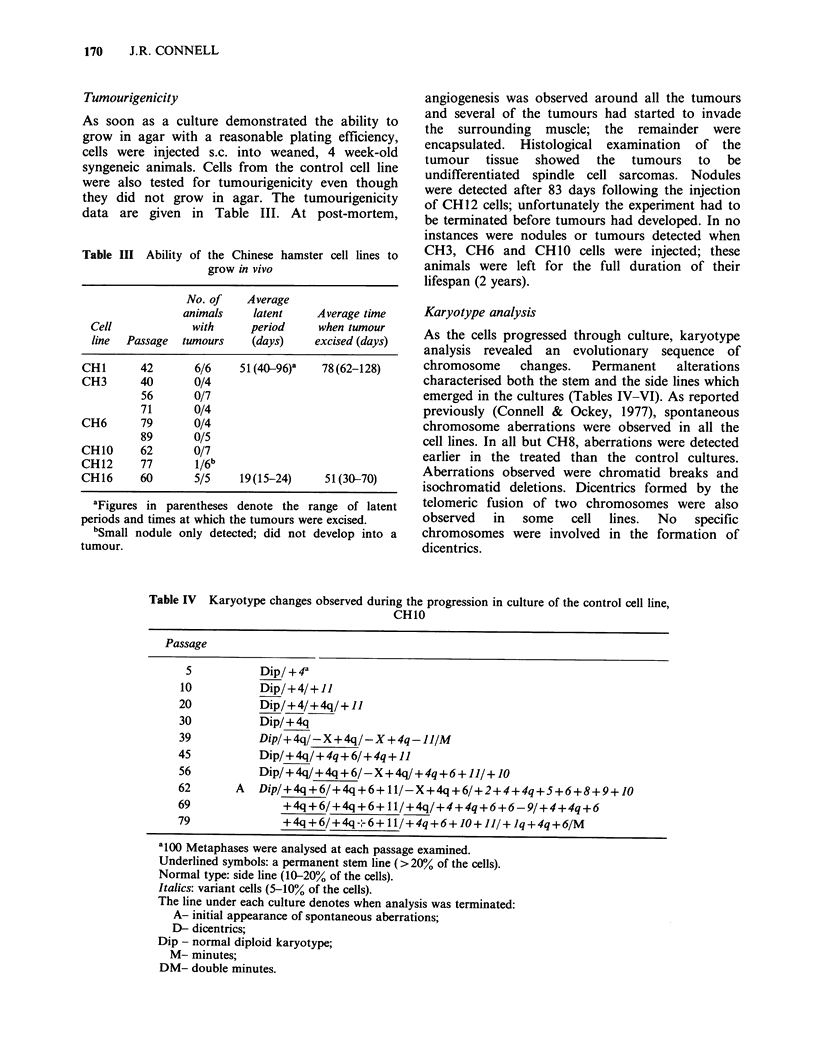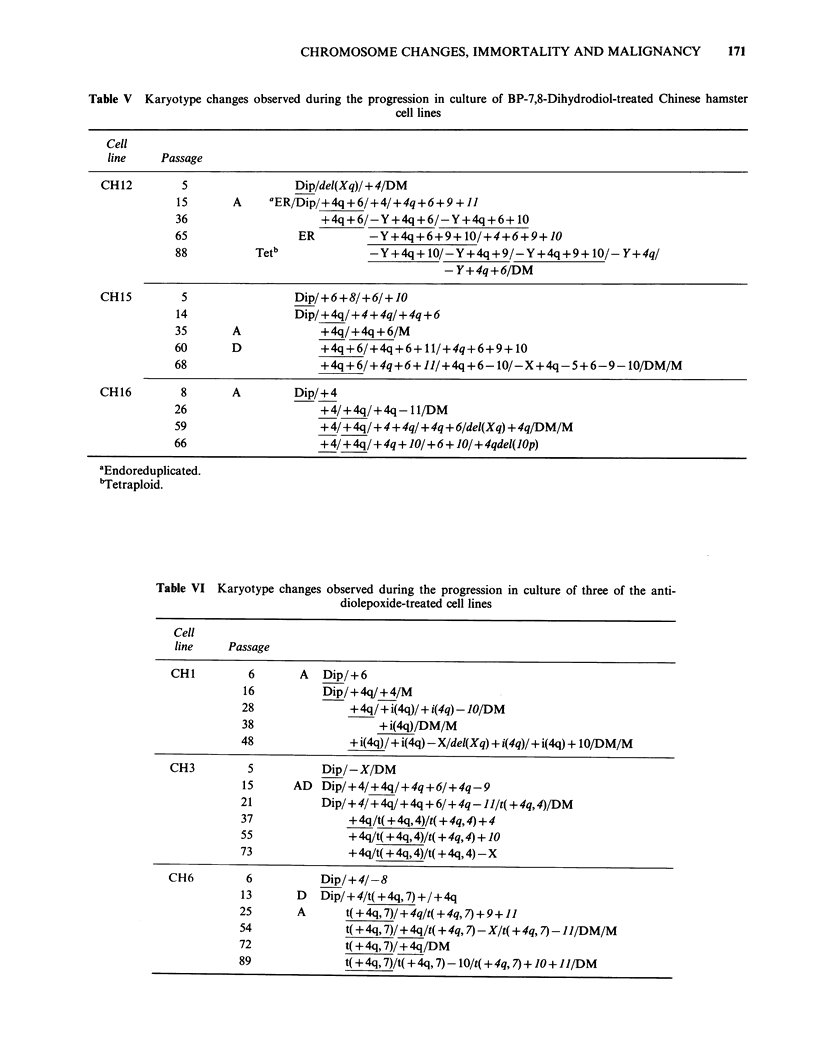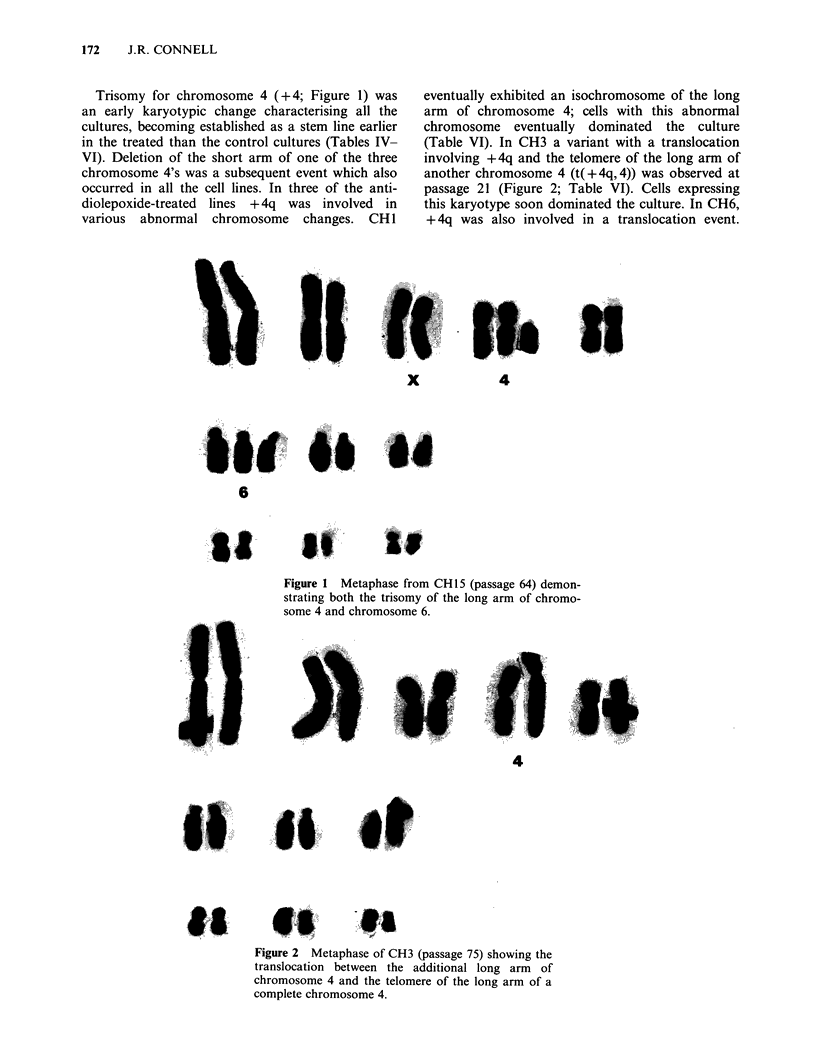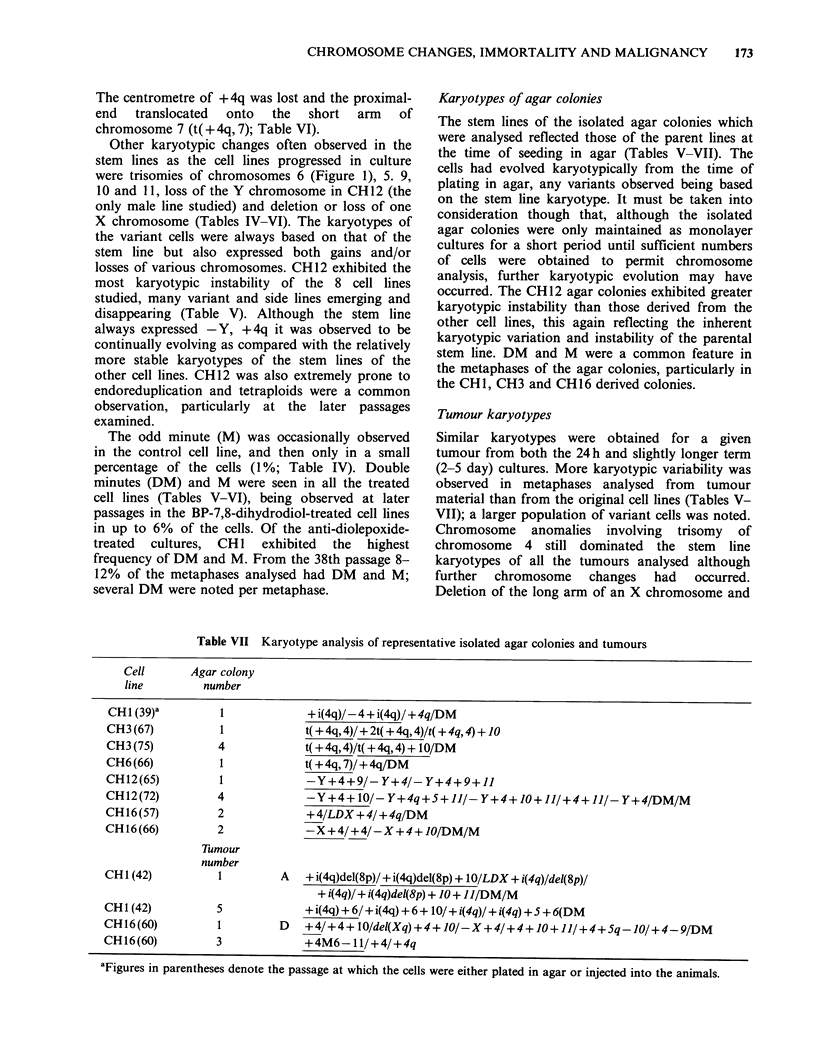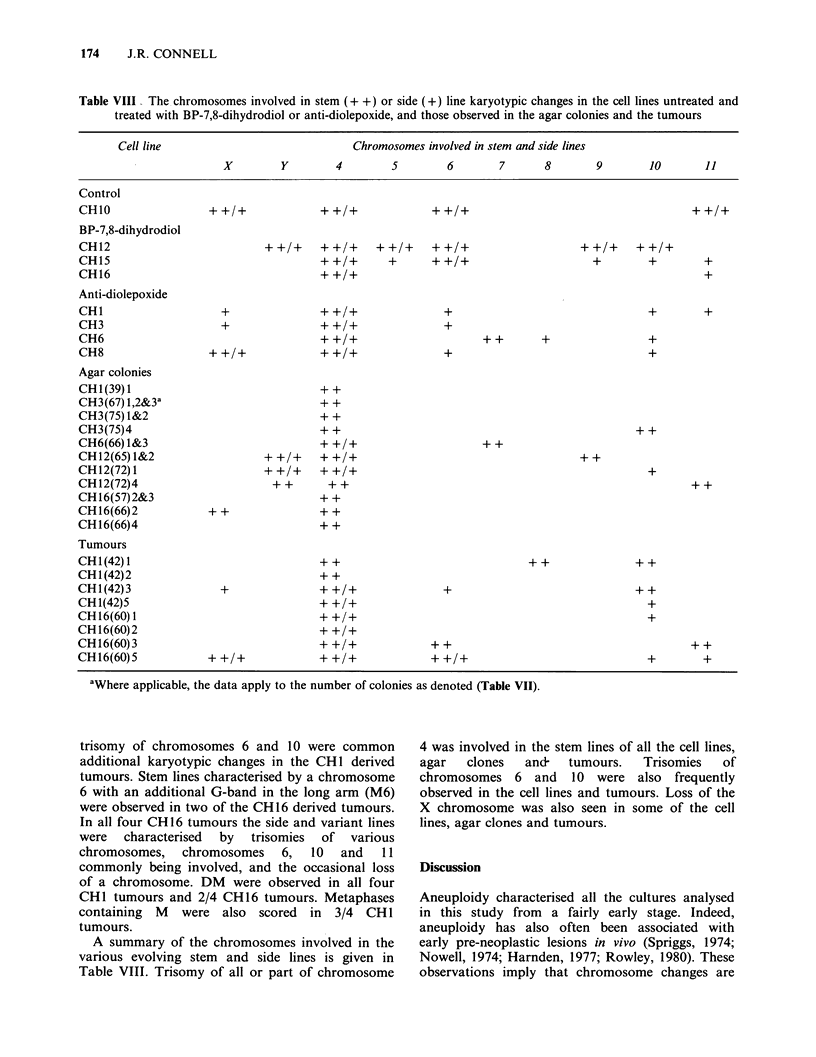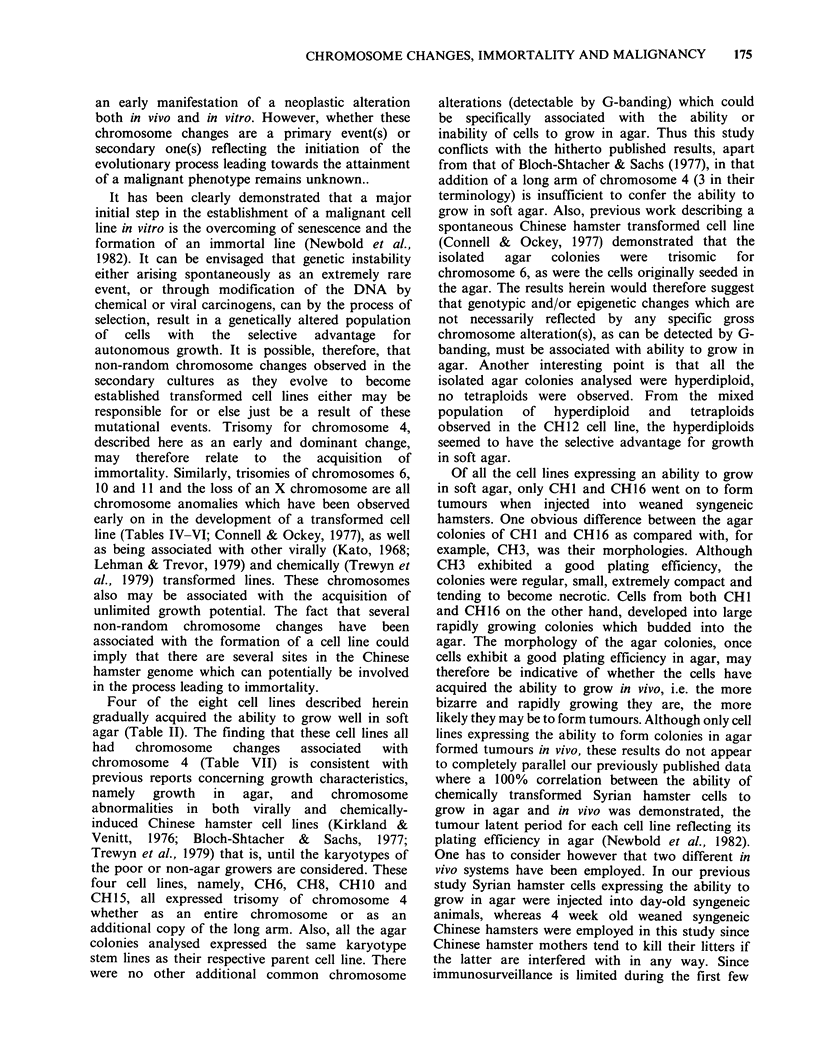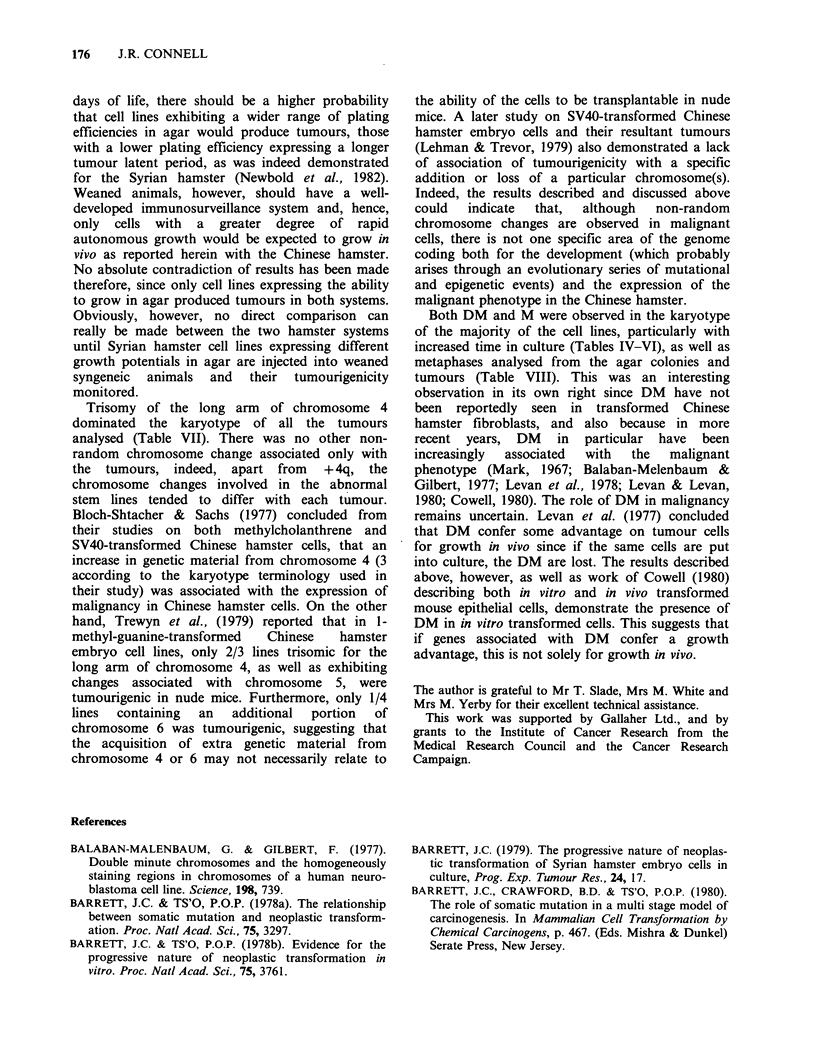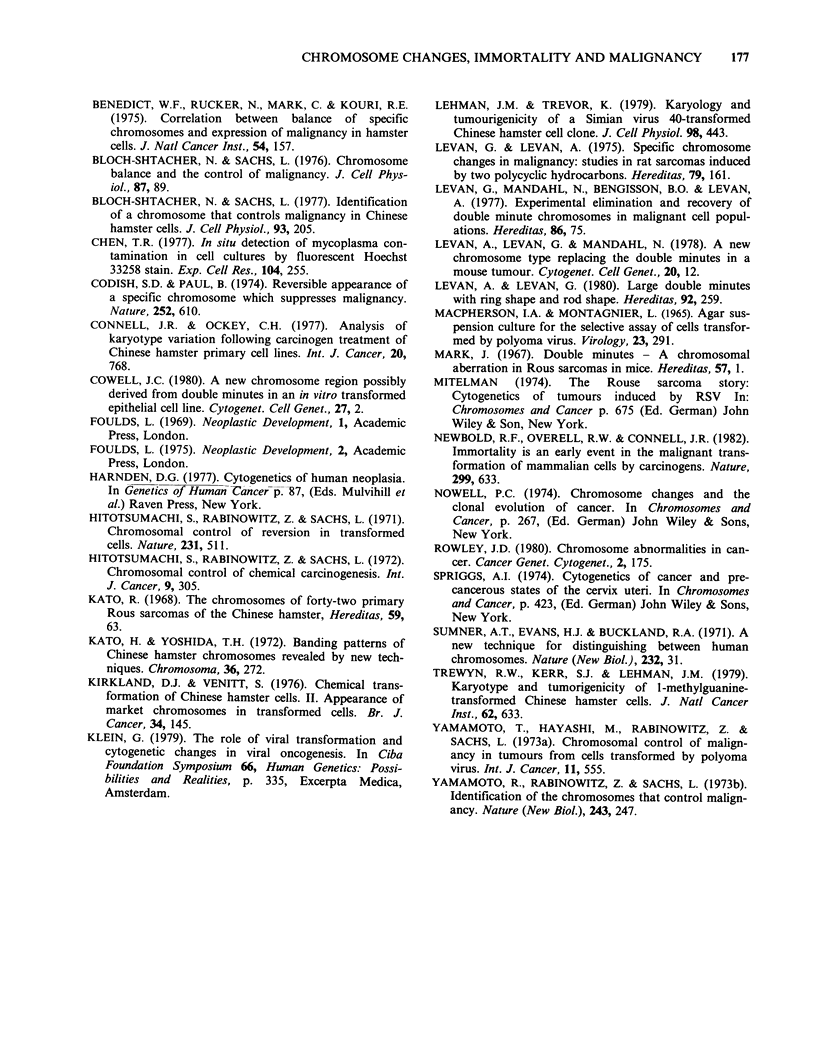# Karyotype analysis of carcinogen-treated Chinese hamster cells in vitro evolving from a normal to a malignant phenotype.

**DOI:** 10.1038/bjc.1984.159

**Published:** 1984-08

**Authors:** J. R. Connell

## Abstract

**Images:**


*Br. J. Cancer* (1984), **50**, 167–177

### Karyotype analysis of carcinogen-treated Chinese hamster cells *in vitro* evolving from a normal to a malignant phenotype

J.R. Connell*

*Chemical Carcinogenesis Division, Institute of Cancer Research, Pollards Wood Research Station, Nightingales Lane, Chalfont St. Giles, Bucks HP8 4SP, UK.*

**Summary** The relationship of cytogenetic changes with the acquisition of an indefinite life span *in vitro*, the ability of cells to grow in soft agar and their tumourigenicity in syngeneic animals has been studied in control, trans-7,8-dihydrodiolbenzo(a)pyrene and 7β,8α-dihydroxy-9α,10α-epoxy-7,8,9,10-tetrahydrobenzo(a)-pyrene-treated secondary cultures derived from Chinese hamster embryonic lung. Karyotype analysis revealed a sequence of chromosome changes as the cells progressed through culture. Aneuploidy, namely trisomy of chromosome 4, the long arm in particular, was an early dominant change. The possible association of this trisomy with the acquisition of immortality *in vitro* is implicated, although the involvement of other non-random chromosome changes cannot be eliminated, implying that there may be several genomic sites in the Chinese hamster which may potentially be involved with the acquisition of unlimited growth potential. Neither the ability of cells to grow in soft agar nor as tumours could be associated with any specific chromosome(s). Double minutes were observed in metaphases from the cell lines, agar colonies and tumours; their possible relationship with growth advantage is discussed.

Karotype abnormalities characterise the majority of metaphases recovered from malignant cells. Indeed, cytogenetic analysis of neoplastic golden hamster cells, revertants with a suppressed malignant phenotype and segregants from these revertants which were again malignant, have led to the identification of chromosomes which control the expression and suppression of malignancy (Hitotsumachi *et al.*, 1971, 1972; Benedict *et al.*, 1975; Bloch-Shtacher & Sachs, 1976). It has also been reported that more than one chromosome may carry genes which control malignancy in this species (Hitotsumachi *et al.*, 1971, 1972; Yamamoto *et al.*, 1973*a*, *b*; Bloch-Shtacher & Sachs, 1976). Some of the factors which control malignancy have also been assigned to specific chromosomes in the rat (Levan & Levan, 1975), mouse (Codish & Paul, 1974; Klein, 1979) and the Chinese hamster (Bloch-Shtacher & Sachs, 1977).

The evolution of the malignant phenotype as a progressive multi-step process has been demonstrated both *in vivo* (Foulds, 1969, 1975) and *in vitro* (Barrett & Ts'o, 1978*a*, *b*; Barrett *et al.*, 1980). *In vitro* studies have indicated that as the neoplastic phenotype evolves, the unknown series of genetic and/or epigenetic events underlying this evolutionary process are reflected through various abnormal phenotypes, i.e. the acquisition of immortality (Newbold *et al.*, 1982) and loss of anchorage dependency for growth (Connell & Ockey, 1977; Barrett, 1979).

*Present address, Agricultural Research Council, Institute of Animal Physiology, Babraham, Cambridge CB2 4AT, UK.

Received 10 November 1983; accepted 19 April 1984.

Many of the aforementioned chromosome studies were made on established neoplastic cells. Since the evolutionary process leading to a malignant phenotype may be monitored *in vitro*, it seemed of value to employ such a system in order to investigate whether any specific chromosome changes could be associated with a particular emergent abnormal phenotype (i.e. the ability to grow in soft agar) and, at what stage in the evolutionary process any chromosome change(s) which have been associated with the expression of malignancy arose. It was decided, therefore, to treat early passage fibroblast cultures derived from Chinese hamster embryonic lung with either trans-7,8-dihydrodiolbenzo(a)pyrene or 7β, 8α-dihydroxy-9α,10α-epoxy-7,8,9,10-tetrahydrobenzo(a)-pyrene the proximal and ultimate carcinogenic metabolites respectively of benzo(a)pyrene. A sequential karyotype analysis was carried out as the cells evolved in culture. The phenotypic changes monitored were the acquisition of immortality, loss of anchorage independence of growth, as determined by the ability of the cells to grow in soft agar, and their tumourigenicity in syngeneic hamsters.

### Materials and methods

Primary cultures were initiated from small fragments of foetal lung pooled from nine Chinese hamster embryos isolated on the 14th day of gestation. The cultures were initiated in 75 $cm^2$ plastic culture flasks (Nunclon, Roskilda, Denmark) in Dulbecco's minimal essential medium (DMEM) supplemented with 10% foetal bovine

© The Macmillan Press Ltd., 1984

serum (Gibco-Europe Ltd., Glasgow, Scotland) and antibiotics. Cultures were maintained at pH 7.2 ± 0.2 by gassing with 10% $CO_2$ in air and were cultured at 37°C. The culture medium (CM) was changed every day until the first subculture. Following the initial subculture the cultures were termed 'secondary cultures'; and when they had obviously overcome senescence and had expressed unlimited growth potential *in vitro*, they were termed 'cell-lines'. The cultures were screened, using the method of Chen (1977), on a regular basis for mycoplasma contamination. The cultures were always mycoplasma free.

*Chemicals*

Trans7,8-dihydrodiolbenzo(a)pyrene(BP7,8-dihydrodiol) and 7$\beta$,8$\alpha$-dihydro-9$\alpha$,10$\alpha$-epoxy-7,8,9,10-tetrahydrobenzo-(a)pyrene(anti-diolepoxide) were provided by Prof. R.G. Harvey, University of Chicago, U.S.A. Stock solutions were prepared in anhydrous dimethylsulphoxide (DMSO) and stored at −20°C.

*Treatment of cells*

The primary cultures were subcultured 1:4 into 75 cm² flasks. Following a 24 h incubation these secondary cultures were treated with 0.1 or 0.5 $\mu$g ml$^{-1}$ BP7, 8-dihydrodiol or anti-diolepoxide (Table I). Controls were treated with the DMSO solvent at 0.4%. Duration of treatment was 24 h. The medium was decanted, the cell monolayer washed with phosphate buffered saline and a fresh volume of CM applied. The medium was changed every 2 days until the first post-treatment subculture (7 days after treatment). The cultures were subcultured 1:4 for the first 10–17 passages after treatment and then 1:10 for the subsequent passages when the rate of exponential growth had increased. Subculturing was carried out every 2–5 days, depending on the rate of growth of the individual cultures, and when the cells were ~75% confluent. Throughout this study, each original treated flask was maintained as an individual culture, thus ensuring that no cross-contamination of cultures occurred.

Karyotype analysis was maintained with time in culture, slide preparations being made at regular intervals. Cells were accumulated at metaphase by treating exponentially growing cultures with 0.05 $\mu$g ml$^{-1}$ colcemid (Gibco-Europe Ltd.) for 2 h. The metaphases were harvested and air-dried slides prepared following hypotonic treatment (0.075 M KCl, 20 min) and fixation in methanol:glacial acetic acid (3:1). Slides were G-banded using a modification of the A.S.G. method (Sumner *et al.*, 1971). Twenty-four hour old slides were incubated in $10^{-6}$% lyophilized trypsin (Sigma London Chemical Co. Ltd., Poole, Dorset) at 37°C for 45–75 min. The slides were then incubated in Hank's basal salt solution (BSS) for 1 h at 60°C, rinsed briefly in deionized water and then in pH 6.8 phosphate buffer, and stained with 2% Giemsa (pH 6.8) for 8–10 min. One hundred metaphases were analysed for each culture at every passage examined. The Kato & Yoshida (1972) nomenclature for the Chinese hamster karyotype was employed. The terminology employed in the karotype analysis was based on that of Mitelman (1974) and is defined in Table IV.

*Growth in soft agar*

The ability of the secondary cultures and the subsequent cell lines derived from these to grow in soft agar was monitored at regular intervals as the cells progressed through culture. The method of Macpherson & Montagnier (1965) was employed. Five ml aliquots of 0.5% Difco Noble agar in CM were introduced into 5 cm vented petri dishes to provide a basal layer. Two ml aliquots of 0.33% agar containing $10^5$ cells were then pipetted on top of the hardened 0.5% agar. The petri dishes were incubated in a gassed, humidified incubator at 37°C for 2–3 weeks. At weekly intervals, 1 ml of CM was gently added to each plate in order to maintain the isotonicity of the agar. Colonies of 50 or more cells were scored and the plating efficiency of the cells in soft agar calculated. Colonies were also isolated from the agar and re-introduced into monolayer culture. Karyotype analysis was carried out as soon as a cell population had increased sufficiently.

*Tumourigenicity*

The ability of the cells to grow *in vivo* was monitored by injecting 0.1 ml sterile saline containing $10^6$ cells subcutaneously into the right thigh of 4 week-old syngeneic Chinese hamsters. Between 4 and 7 animals were used in each experiment. The animals were examined thrice weekly for tumour growth. The latent period for tumour development was taken at the time when the tumours first became palpable. When tumours did arise they were excised. Pieces of tumour tissue were fixed in Bouin's and then prepared for histology. The tissue sections were stained with haematoxylin and eosin. The remaining tumour tissue was put into culture. Karyotype analysis was carried out both on short-term (24 h) cultures and at the first passage following the initiation of the tumours in culture. Metaphases were harvested from short-term cultures by placing macerated tumour in CM containing 0.05 $\mu$g ml$^{-1}$ colcemid for 24 h and then harvesting for slide preparations. The protocol followed for making the slide preparations and G-banding were as described above.

### Results

Eighteen secondary cultures were originally treated with the two chemicals, only 7 however went on to form continuous cell lines. Out of the 10 control flasks only one cell line emerged, CH10. The remaining 20 treated and control cultures senesced between passages 12 and 20 when a decline in growth rate was observed, this leading to a complete loss of division potential. The cells also became greatly enlarged and flattened in morphology. Of the 8 cell lines, only CH12 was observed to go through an obvious 'crisis' at the end of the proliferative phase of growth (passage 14) before immortal cells appeared, these giving rise to a continuous cell line. With the remaining secondary cultures no crisis was observed indicating that immortal cells had emerged prior to the cessation of normal cell division. The work reported herein describes an analysis of these 8 cultures as they progressed through culture. The carcinogen treatment and the prefixes given to these lines are shown in Table I.

**Table I** Carcinogen treatment administered to the secondary cultures and the prefixes given to each culture

| *Treatment* ($\mu g\, ml^{-1}$) | *Culture prefix* |
|---|---|
| 0 | CH10 |
| BP-7,8-dihydrodiol | |
| 0.1 | CH12 |
| 0.5 | CH15 |
| 0.5 | CH16 |
| Anti-diolepoxide | |
| 0.1 | CH1 |
| 0.1 | CH8 |
| 0.5 | CH3 |
| 0.5 | CH6 |

The morphology of the cell cultures at the time of treatment was that of a typical fibroblast culture. Strong contact inhibition of growth and parallel orientation was observed. From the 8th week in culture the BP-7,8-dihydrodiol-treated CH12 and 16 cultures and the anti-diolepoxide cultures CH1 and 8 gradually attained a more cuboidal morphology. The control, CH10, retained a normal fibroblastic morphology throughout the period of culture, whereas CH3, 6 and 15 became more spindle shaped and demonstrated piling up and loss of orientation of the cells.

### *Growth in soft agar*

As the secondary cultures progressed through culture they were routinely monitored for their ability to grow in soft agar. The results pertaining to these experiments are shown in Table II. CH10 was never observed to grow in agar, the majority of cells dying after 7–10 days in agar and only a few viable single cells were observed. Several of the treated cultures however gradually acquired the ability to grow in agar. CH1 demonstrated the highest plating efficiency. Large and viable colonies with cells budding in a stranded fashion into the agar were observed. CH3 also grew in agar but, even at later passages when the plating efficiency had improved, the colonies were smaller and more compact than those of CH1 and the centre of many colonies appeared necrotic. CH6, 8 and 15 demonstrated a limited ability to grow in agar; the morphology of these colonies was similar to those of CH3. CH12 gradually acquired a moderate plating efficiency in agar. By passage 72 the colonies were large and budding into the agar as observed with CH1. At passage 86, not shown in Table II, the plating efficiency had increased such that an accurate count could not be made (>1,500 colonies/plate). As with CH12, CH16 demonstrated a gradual increase in plating efficiency and by passage 66 large budding colonies were observed.

**Table II** The ability of the various cell lines to grow in soft agar

| *Cell line* | *Passage number* | *Average no. of colonies* | [a]*Plating efficiency* (%) *in agar* (±s.d.) |
|---|---|---|---|
| CH1 | 36 | 0 | 0 |
| | 39 | 1425 | 1.43 (±0.1) |
| CH3 | 35 | 0 | 0 |
| | 38 | 33 | 0.033 (±0.004) |
| | 42 | 318 | 0.31 (±0.04) |
| | 67 | 235 | 0.25 (±0.002) |
| CH6 | 41 | 1 | 0.0075 (±0.00027) |
| | 74 | 19 | 0.019 (±0.006) |
| | 88 | 31 | 0.03 (±0.009) |
| CH8 | 34 | 0 | 0 |
| | 36 | 16 | 0.016 (±0.002) |
| | 41 | 43 | 0.044 (±0.001) |
| CH12 | 57 | 2 | 0.0016 (±0.0001) |
| | 65 | 29 | 0.027 (±0.007) |
| | 72 | 227 | 0.23 (±0.07) |
| CH10 | 36 | 0 | 0 |
| | 57 | 0 | 0 |
| | 76 | 0 | 0 |
| CH15 | 31 | 0 | 0 |
| | 33 | 39 | 0.039 (±0.002) |
| | 55 | 2 | 0.0018 (±0.0012) |
| CH16 | 34 | 0 | 0 |
| | 37 | 29 | 0.029 (±0.002) |
| | 57 | 31 | 0.033 (±0.005) |
| | 66 | 238 | 0.234 (±0.04) |

[a]$10^5$ plated/petri dish.

*Tumourigenicity*

As soon as a culture demonstrated the ability to grow in agar with a reasonable plating efficiency, cells were injected s.c. into weaned, 4 week-old syngeneic animals. Cells from the control cell line were also tested for tumourigenicity even though they did not grow in agar. The tumourigenicity data are given in Table III. At post-mortem, angiogenesis was observed around all the tumours and several of the tumours had started to invade the surrounding muscle; the remainder were encapsulated. Histological examination of the tumour tissue showed the tumours to be undifferentiated spindle cell sarcomas. Nodules were detected after 83 days following the injection of CH12 cells; unfortunately the experiment had to be terminated before tumours had developed. In no instances were nodules or tumours detected when CH3, CH6 and CH10 cells were injected; these animals were left for the full duration of their lifespan (2 years).

**Table III** Ability of the Chinese hamster cell lines to grow *in vivo*

| *Cell line* | *Passage* | *No. of animals with tumours* | *Average latent period (days)* | *Average time when tumour excised (days)* |
|---|---|---|---|---|
| CH1 | 42 | 6/6 | 51 (40–96)[a] | 78 (62–128) |
| CH3 | 40 | 0/4 | | |
| | 56 | 0/7 | | |
| | 71 | 0/4 | | |
| CH6 | 79 | 0/4 | | |
| | 89 | 0/5 | | |
| CH10 | 62 | 0/7 | | |
| CH12 | 77 | 1/6[b] | | |
| CH16 | 60 | 5/5 | 19 (15–24) | 51 (30–70) |

[a]Figures in parentheses denote the range of latent periods and times at which the tumours were excised.

[b]Small nodule only detected; did not develop into a tumour.

*Karyotype analysis*

As the cells progressed through culture, karyotype analysis revealed an evolutionary sequence of chromosome changes. Permanent alterations characterised both the stem and the side lines which emerged in the cultures (Tables IV–VI). As reported previously (Connell & Ockey, 1977), spontaneous chromosome aberrations were observed in all the cell lines. In all but CH8, aberrations were detected earlier in the treated than the control cultures. Aberrations observed were chromatid breaks and isochromatid deletions. Dicentrics formed by the telomeric fusion of two chromosomes were also observed in some cell lines. No specific chromosomes were involved in the formation of dicentrics.

**Table IV** Karyotype changes observed during the progression in culture of the control cell line, CH10

| *Passage* | | |
|---|---|---|
| 5 | | Dip/+*4*[a] |
| 10 | | Dip/+4/+*11* |
| 20 | | Dip/+4/+4q/+*11* |
| 30 | | Dip/+4q |
| 39 | | *Dip*/+4q/−X+4q/−*X+4q−11/M* |
| 45 | | Dip/+4q/+*4q*+6/+*4q*+*11* |
| 56 | | Dip/+4q/+4q+6/−X+4q/+*4q*+*6*+*11*/+*10* |
| 62 | A | *Dip*/+4q+6/+4q+6+11/−X+4q+6/+*2*+*4*+*4q*+*5*+*6*+*8*+*9*+*10* |
| 69 | | +4q+6/+4q+6+11/+4q/+*4*+*4q*+*6*+*6*−*9*/+*4*+*4q*+*6* |
| 79 | | +4q+6/+4q÷6+11/+*4q*+*6*+*10*+*11*/+*1q*+*4q*+*6*/M |

[a]100 Metaphases were analysed at each passage examined.
Underlined symbols: a permanent stem line (>20% of the cells).
Normal type: side line (10–20% of the cells).
*Italics*: variant cells (5–10% of the cells).
The line under each culture denotes when analysis was terminated:
A– initial appearance of spontaneous aberrations;
D– dicentrics;
Dip – normal diploid karyotype;
M– minutes;
DM– double minutes.

**Table V** Karyotype changes observed during the progression in culture of BP-7,8-Dihydrodiol-treated Chinese hamster cell lines

| *Cell line* | *Passage* | | |
|---|---|---|---|
| CH12 | 5 | | Dip/*del*(*Xq*)/+*4*/DM |
| | 15 | A | [a]ER/Dip/+4q+6/+4/+*4q*+*6*+*9*+*11* |
| | 36 | | +4q+6/−Y+4q+6/−Y+4q+6+10 |
| | 65 | ER | −Y+4q+6+9+10/+*4*+*6*+*9*+*10* |
| | 88 | Tet[b] | −Y+4q+10/−Y+4q+9/−Y+4q+9+10/−*Y*+*4q*/−*Y*+*4q*+*6*/DM |
| CH15 | 5 | | Dip/+*6*+*8*/+*6*/+*10* |
| | 14 | | Dip/+4q/+*4*+*4q*/+*4q*+*6* |
| | 35 | A | +4q/+4q+6/M |
| | 60 | D | +4q+6/+4q+6+11/+*4q*+6+9+10 |
| | 68 | | +4q+6/+*4q*+*6*+*11*/+4q+6−10/−X+4q−5+6−9−10/DM/M |
| CH16 | 8 | A | Dip/+4 |
| | 26 | | +4/+4q/+4q−11/DM |
| | 59 | | +4/+4q/+*4*+*4q*/+*4q*+*6*/*del*(*Xq*)+*4q*/DM/M |
| | 66 | | +4/+4q/+*4q*+*10*/+*6*+*10*/+*4qdel*(*10p*) |

[a]Endoreduplicated.
[b]Tetraploid.

**Table VI** Karyotype changes observed during the progression in culture of three of the anti-diolepoxide-treated cell lines

| *Cell line* | *Passage* | | |
|---|---|---|---|
| CH1 | 6 | A | Dip/+6 |
| | 16 | | Dip/+4q/+4/M |
| | 28 | | +4q/+i(4q)/+*i*(*4q*)−*10*/DM |
| | 38 | | +i(4q)/DM/M |
| | 48 | | +i(4q)/+i(4q)−X/*del*(*Xq*)+*i*(*4q*)/+i(4q)+10/DM/M |
| CH3 | 5 | | Dip/−*X*/DM |
| | 15 | AD | Dip/+4/+4q/+*4q*+*6*/+*4q*−*9* |
| | 21 | | Dip/+*4*/+4q/+4q+6/+*4q*−*11*/*t*(+*4q*,*4*)/DM |
| | 37 | | +4q/t(+4q,4)/*t*(+*4q*,*4*)+*4* |
| | 55 | | +4q/t(+4q,4)/*t*(+*4q*,*4*)+*10* |
| | 73 | | +4q/t(+4q,4)/t(+4q,4)−X |
| CH6 | 6 | | Dip/+*4*/−*8* |
| | 13 | D | Dip/+*4*/t(+4q,7)+/+4q |
| | 25 | A | t(+4q,7)/+*4q*/*t*(+*4q*,*7*)+*9*+*11* |
| | 54 | | t(+4q,7)/+4q/*t*(+*4q*,*7*)−*X*/*t*(+*4q*,*7*)−*11*/DM/M |
| | 72 | | t(+4q,7)/+4q/DM |
| | 89 | | t(+4q,7)/t(+4q,7)−10/*t*(+*4q*,*7*)+*10*+*11*/DM |

Trisomy for chromosome 4 (+4; Figure 1) was an early karyotypic change characterising all the cultures, becoming established as a stem line earlier in the treated than the control cultures (Tables IV–VI). Deletion of the short arm of one of the three chromosome 4's was a subsequent event which also occurred in all the cell lines. In three of the anti-diolepoxide-treated lines +4q was involved in various abnormal chromosome changes. CH1 eventually exhibited an isochromosome of the long arm of chromosome 4; cells with this abnormal chromosome eventually dominated the culture (Table VI). In CH3 a variant with a translocation involving +4q and the telomere of the long arm of another chromosome 4 (t(+4q, 4)) was observed at passage 21 (Figure 2; Table VI). Cells expressing this karyotype soon dominated the culture. In CH6, +4q was also involved in a translocation event.

**Figure 1** Metaphase from CH15 (passage 64) demonstrating both the trisomy of the long arm of chromosome 4 and chromosome 6.

**Figure 2** Metaphase of CH3 (passage 75) showing the translocation between the additional long arm of chromosome 4 and the telomere of the long arm of a complete chromosome 4.

The centrometre of +4q was lost and the proximal-end translocated onto the short arm of chromosome 7 (t(+4q, 7); Table VI).

Other karyotypic changes often observed in the stem lines as the cell lines progressed in culture were trisomies of chromosomes 6 (Figure 1), 5. 9, 10 and 11, loss of the Y chromosome in CH12 (the only male line studied) and deletion or loss of one X chromosome (Tables IV–VI). The karyotypes of the variant cells were always based on that of the stem line but also expressed both gains and/or losses of various chromosomes. CH12 exhibited the most karyotypic instability of the 8 cell lines studied, many variant and side lines emerging and disappearing (Table V). Although the stem line always expressed −Y, +4q it was observed to be continually evolving as compared with the relatively more stable karyotypes of the stem lines of the other cell lines. CH12 was also extremely prone to endoreduplication and tetraploids were a common observation, particularly at the later passages examined.

The odd minute (M) was occasionally observed in the control cell line, and then only in a small percentage of the cells (1%; Table IV). Double minutes (DM) and M were seen in all the treated cell lines (Tables V–VI), being observed at later passages in the BP-7,8-dihydrodiol-treated cell lines in up to 6% of the cells. Of the anti-diolepoxide-treated cultures, CH1 exhibited the highest frequency of DM and M. From the 38th passage 8–12% of the metaphases analysed had DM and M; several DM were noted per metaphase.

### *Karyotypes of agar colonies*

The stem lines of the isolated agar colonies which were analysed reflected those of the parent lines at the time of seeding in agar (Tables V–VII). The cells had evolved karyotypically from the time of plating in agar, any variants observed being based on the stem line karyotype. It must be taken into consideration though that, although the isolated agar colonies were only maintained as monolayer cultures for a short period until sufficient numbers of cells were obtained to permit chromosome analysis, further karyotypic evolution may have occurred. The CH12 agar colonies exhibited greater karyotypic instability than those derived from the other cell lines, this again reflecting the inherent karyotypic variation and instability of the parental stem line. DM and M were a common feature in the metaphases of the agar colonies, particularly in the CH1, CH3 and CH16 derived colonies.

### *Tumour karyotypes*

Similar karyotypes were obtained for a given tumour from both the 24 h and slightly longer term (2–5 day) cultures. More karyotypic variability was observed in metaphases analysed from tumour material than from the original cell lines (Tables V–VII); a larger population of variant cells was noted. Chromosome anomalies involving trisomy of chromosome 4 still dominated the stem line karyotypes of all the tumours analysed although further chromosome changes had occurred. Deletion of the long arm of an X chromosome and

**Table VII** Karyotype analysis of representative isolated agar colonies and tumours

| *Cell line* | *Agar colony number* | | |
|---|---|---|---|
| CH1 (39)[a] | 1 | | +i(4q)/−4+i(4q)/+*4q*/DM |
| CH3 (67) | 1 | | t(+4q, 4)/+2t(+4q, 4)/*t*(+*4q*, *4*)+*10* |
| CH3 (75) | 4 | | t(+4q, 4)/t(+4q, 4)+10/DM |
| CH6 (66) | 1 | | t(+4q, 7)/+4q/DM |
| CH12 (65) | 1 | | −Y+4+9/−*Y*+*4*/−*Y*+*4*+*9*+*11* |
| CH12 (72) | 4 | | −Y+4+10/−*Y*+*4q*+*5*+*11*/−*Y*+*4*+*10*+*11*/+*4*+*11*/−*Y*+*4*/DM/M |
| CH16 (57) | 2 | | +4/*LDX*+*4*/+*4q*/DM |
| CH16 (66) | 2 | | −X+4/+4/−*X*+*4*+*10*/DM/M |
| | *Tumour number* | | |
| CH1 (42) | 1 | A | +i(4q)del(8p)/+i(4q)del(8p)+10/*LDX*+*i*(*4q*)/*del*(8*p*)/ +*i*(*4q*)/+*i*(4*q*)*del*(*8p*)+*10*+*11*/DM/M |
| CH1 (42) | 5 | | +i(4q)+6/+i(4q)+6+10/+*i*(*4q*)/+*i*(*4q*)+*5*+*6*(DM |
| CH16 (60) | 1 | D | +4/+4+10/*del*(*Xq*)+*4*+*10*/−*X*+*4*/+*4*+*10*+*11*/+*4*+*5q*−*10*/+*4*−*9*/DM |
| CH16 (60) | 3 | | +4M6−11/+4/+*4q* |

[a]Figures in parentheses denote the passage at which the cells were either plated in agar or injected into the animals.

**Table VIII**. The chromosomes involved in stem (+ +) or side (+) line karyotypic changes in the cell lines untreated and treated with BP-7,8-dihydrodiol or anti-diolepoxide, and those observed in the agar colonies and the tumours

| *Cell line* | *Chromosomes involved in stem and side lines* | | | | | | | | | |
|---|---|---|---|---|---|---|---|---|---|---|
| | *X* | *Y* | *4* | *5* | *6* | *7* | *8* | *9* | *10* | *11* |
| Control | | | | | | | | | | |
| CH10 | + +/+ | | + +/+ | | + +/+ | | | | | + +/+ |
| BP-7,8-dihydrodiol | | | | | | | | | | |
| CH12 | | + +/+ | + +/+ | + +/+ | + +/+ | | | + +/+ | + +/+ | |
| CH15 | | | + +/+ | + | + +/+ | | | + | + | + |
| CH16 | | | + +/+ | | | | | | | + |
| Anti-diolepoxide | | | | | | | | | | |
| CH1 | + | | + +/+ | | + | | | | + | + |
| CH3 | + | | + +/+ | | + | | | | | |
| CH6 | | | + +/+ | | | + + | + | | + | |
| CH8 | + +/+ | | + +/+ | | + | | | | + | |
| Agar colonies | | | | | | | | | | |
| CH1(39)1 | | | + + | | | | | | | |
| CH3(67)1,2&3[a] | | | + + | | | | | | | |
| CH3(75)1&2 | | | + + | | | | | | | |
| CH3(75)4 | | | + + | | | | | | + + | |
| CH6(66)1&3 | | | + +/+ | | | + + | | | | |
| CH12(65)1&2 | | + +/+ | + +/+ | | | | | + + | | |
| CH12(72)1 | | + +/+ | + +/+ | | | | | | + | |
| CH12(72)4 | | + + | + + | | | | | | | + + |
| CH16(57)2&3 | | | + + | | | | | | | |
| CH16(66)2 | + + | | + + | | | | | | | |
| CH16(66)4 | | | + + | | | | | | | |
| Tumours | | | | | | | | | | |
| CH1(42)1 | | | + + | | | | + + | | + + | |
| CH1(42)2 | | | + + | | | | | | | |
| CH1(42)3 | + | | + +/+ | | + | | | | + + | |
| CH1(42)5 | | | + +/+ | | | | | | + | |
| CH16(60)1 | | | + +/+ | | | | | | + | |
| CH16(60)2 | | | + +/+ | | | | | | | |
| CH16(60)3 | | | + +/+ | | + + | | | | | + + |
| CH16(60)5 | + +/+ | | + +/+ | | + +/+ | | | | + | + |

[a]Where applicable, the data apply to the number of colonies as denoted (**Table VII**).

trisomy of chromosomes 6 and 10 were common additional karyotypic changes in the CH1 derived tumours. Stem lines characterised by a chromosome 6 with an additional G-band in the long arm (M6) were observed in two of the CH16 derived tumours. In all four CH16 tumours the side and variant lines were characterised by trisomies of various chromosomes, chromosomes 6, 10 and 11 commonly being involved, and the occasional loss of a chromosome. DM were observed in all four CH1 tumours and 2/4 CH16 tumours. Metaphases containing M were also scored in 3/4 CH1 tumours.

A summary of the chromosomes involved in the various evolving stem and side lines is given in Table VIII. Trisomy of all or part of chromosome 4 was involved in the stem lines of all the cell lines, agar clones and tumours. Trisomies of chromosomes 6 and 10 were also frequently observed in the cell lines and tumours. Loss of the X chromosome was also seen in some of the cell lines, agar clones and tumours.

### Discussion

Aneuploidy characterised all the cultures analysed in this study from a fairly early stage. Indeed, aneuploidy has also often been associated with early pre-neoplastic lesions *in vivo* (Spriggs, 1974; Nowell, 1974; Harnden, 1977; Rowley, 1980). These observations imply that chromosome changes are an early manifestation of a neoplastic alteration both *in vivo* and *in vitro*. However, whether these chromosome changes are a primary event(s) or secondary one(s) reflecting the initiation of the evolutionary process leading towards the attainment of a malignant phenotype remains unknown..

It has been clearly demonstrated that a major initial step in the establishment of a malignant cell line *in vitro* is the overcoming of senescence and the formation of an immortal line (Newbold *et al.*, 1982). It can be envisaged that genetic instability either arising spontaneously as an extremely rare event, or through modification of the DNA by chemical or viral carcinogens, can by the process of selection, result in a genetically altered population of cells with the selective advantage for autonomous growth. It is possible, therefore, that non-random chromosome changes observed in the secondary cultures as they evolve to become established transformed cell lines either may be responsible for or else just be a result of these mutational events. Trisomy for chromosome 4, described here as an early and dominant change, may therefore relate to the acquisition of immortality. Similarly, trisomies of chromosomes 6, 10 and 11 and the loss of an X chromosome are all chromosome anomalies which have been observed early on in the development of a transformed cell line (Tables IV–VI; Connell & Ockey, 1977), as well as being associated with other virally (Kato, 1968; Lehman & Trevor, 1979) and chemically (Trewyn *et al.*, 1979) transformed lines. These chromosomes also may be associated with the acquisition of unlimited growth potential. The fact that several non-random chromosome changes have been associated with the formation of a cell line could imply that there are several sites in the Chinese hamster genome which can potentially be involved in the process leading to immortality.

Four of the eight cell lines described herein gradually acquired the ability to grow well in soft agar (Table II). The finding that these cell lines all had chromosome changes associated with chromosome 4 (Table VII) is consistent with previous reports concerning growth characteristics, namely growth in agar, and chromosome abnormalities in both virally and chemically-induced Chinese hamster cell lines (Kirkland & Venitt, 1976; Bloch-Shtacher & Sachs, 1977; Trewyn *et al.*, 1979) that is, until the karyotypes of the poor or non-agar growers are considered. These four cell lines, namely, CH6, CH8, CH10 and CH15, all expressed trisomy of chromosome 4 whether as an entire chromosome or as an additional copy of the long arm. Also, all the agar colonies analysed expressed the same karyotype stem lines as their respective parent cell line. There were no other additional common chromosome alterations (detectable by G-banding) which could be specifically associated with the ability or inability of cells to grow in agar. Thus this study conflicts with the hitherto published results, apart from that of Bloch-Shtacher & Sachs (1977), in that addition of a long arm of chromosome 4 (3 in their terminology) is insufficient to confer the ability to grow in soft agar. Also, previous work describing a spontaneous Chinese hamster transformed cell line (Connell & Ockey, 1977) demonstrated that the isolated agar colonies were trisomic for chromosome 6, as were the cells originally seeded in the agar. The results herein would therefore suggest that genotypic and/or epigenetic changes which are not necessarily reflected by any specific gross chromosome alteration(s), as can be detected by G-banding, must be associated with ability to grow in agar. Another interesting point is that all the isolated agar colonies analysed were hyperdiploid, no tetraploids were observed. From the mixed population of hyperdiploid and tetraploids observed in the CH12 cell line, the hyperdiploids seemed to have the selective advantage for growth in soft agar.

Of all the cell lines expressing an ability to grow in soft agar, only CH1 and CH16 went on to form tumours when injected into weaned syngeneic hamsters. One obvious difference between the agar colonies of CH1 and CH16 as compared with, for example, CH3, was their morphologies. Although CH3 exhibited a good plating efficiency, the colonies were regular, small, extremely compact and tending to become necrotic. Cells from both CH1 and CH16 on the other hand, developed into large rapidly growing colonies which budded into the agar. The morphology of the agar colonies, once cells exhibit a good plating efficiency in agar, may therefore be indicative of whether the cells have acquired the ability to grow *in vivo*, i.e. the more bizarre and rapidly growing they are, the more likely they may be to form tumours. Although only cell lines expressing the ability to form colonies in agar formed tumours *in vivo*, these results do not appear to completely parallel our previously published data where a 100% correlation between the ability of chemically transformed Syrian hamster cells to grow in agar and *in vivo* was demonstrated, the tumour latent period for each cell line reflecting its plating efficiency in agar (Newbold *et al.*, 1982). One has to consider however that two different *in vivo* systems have been employed. In our previous study Syrian hamster cells expressing the ability to grow in agar were injected into day-old syngeneic animals, whereas 4 week old weaned syngeneic Chinese hamsters were employed in this study since Chinese hamster mothers tend to kill their litters if the latter are interfered with in any way. Since immunosurveillance is limited during the first few days of life, there should be a higher probability that cell lines exhibiting a wider range of plating efficiencies in agar would produce tumours, those with a lower plating efficiency expressing a longer tumour latent period, as was indeed demonstrated for the Syrian hamster (Newbold *et al.*, 1982). Weaned animals, however, should have a well-developed immunosurveillance system and, hence, only cells with a greater degree of rapid autonomous growth would be expected to grow *in vivo* as reported herein with the Chinese hamster. No absolute contradiction of results has been made therefore, since only cell lines expressing the ability to grow in agar produced tumours in both systems. Obviously, however, no direct comparison can really be made between the two hamster systems until Syrian hamster cell lines expressing different growth potentials in agar are injected into weaned syngeneic animals and their tumourigenicity monitored.

Trisomy of the long arm of chromosome 4 dominated the karyotype of all the tumours analysed (Table VII). There was no other non-random chromosome change associated only with the tumours, indeed, apart from +4q, the chromosome changes involved in the abnormal stem lines tended to differ with each tumour. Bloch-Shtacher & Sachs (1977) concluded from their studies on both methylcholanthrene and SV40-transformed Chinese hamster cells, that an increase in genetic material from chromosome 4 (3 according to the karyotype terminology used in their study) was associated with the expression of malignancy in Chinese hamster cells. On the other hand, Trewyn *et al.*, (1979) reported that in 1-methyl-guanine-transformed Chinese hamster embryo cell lines, only 2/3 lines trisomic for the long arm of chromosome 4, as well as exhibiting changes associated with chromosome 5, were tumourigenic in nude mice. Furthermore, only 1/4 lines containing an additional portion of chromosome 6 was tumourigenic, suggesting that the acquisition of extra genetic material from chromosome 4 or 6 may not necessarily relate to the ability of the cells to be transplantable in nude mice. A later study on SV40-transformed Chinese hamster embryo cells and their resultant tumours (Lehman & Trevor, 1979) also demonstrated a lack of association of tumourigenicity with a specific addition or loss of a particular chromosome(s). Indeed, the results described and discussed above could indicate that, although non-random chromosome changes are observed in malignant cells, there is not one specific area of the genome coding both for the development (which probably arises through an evolutionary series of mutational and epigenetic events) and the expression of the malignant phenotype in the Chinese hamster.

Both DM and M were observed in the karyotype of the majority of the cell lines, particularly with increased time in culture (Tables IV–VI), as well as metaphases analysed from the agar colonies and tumours (Table VIII). This was an interesting observation in its own right since DM have not been reportedly seen in transformed Chinese hamster fibroblasts, and also because in more recent years, DM in particular have been increasingly associated with the malignant phenotype (Mark, 1967; Balaban-Melenbaum & Gilbert, 1977; Levan *et al.*, 1978; Levan & Levan, 1980; Cowell, 1980). The role of DM in malignancy remains uncertain. Levan *et al.* (1977) concluded that DM confer some advantage on tumour cells for growth *in vivo* since if the same cells are put into culture, the DM are lost. The results described above, however, as well as work of Cowell (1980) describing both *in vitro* and *in vivo* transformed mouse epithelial cells, demonstrate the presence of DM in *in vitro* transformed cells. This suggests that if genes associated with DM confer a growth advantage, this is not solely for growth *in vivo*.

The author is grateful to Mr T. Slade, Mrs M. White and Mrs M. Yerby for their excellent technical assistance.

This work was supported by Gallaher Ltd., and by grants to the Institute of Cancer Research from the Medical Research Council and the Cancer Research Campaign.

### References

BALABAN-MALENBAUM, G. & GILBERT, F. (1977). Double minute chromosomes and the homogeneously staining regions in chromosomes of a human neuroblastoma cell line. *Science*, **198**, 739.

BARRETT, J.C. & TS'O, P.O.P. (1978a). The relationship between somatic mutation and neoplastic transformation. *Proc. Natl Acad. Sci.*, **75**, 3297.

BARRETT, J.C. & TS'O, P.O.P. (1978b). Evidence for the progressive nature of neoplastic transformation *in vitro*. *Proc. Natl Acad. Sci.*, **75**, 3761.

BARRETT, J.C. (1979). The progressive nature of neoplastic transformation of Syrian hamster embryo cells in culture, *Prog. Exp. Tumour Res.*, **24**, 17.

BARRETT, J.C., CRAWFORD, B.D. & TS'O, P.O.P. (1980). The role of somatic mutation in a multi stage model of carcinogenesis. In *Mammalian Cell Transformation by Chemical Carcinogens*, p. 467. (Eds. Mishra & Dunkel) Serate Press, New Jersey.

BENEDICT, W.F., RUCKER, N., MARK, C. & KOURI, R.E. (1975). Correlation between balance of specific chromosomes and expression of malignancy in hamster cells. *J. Natl Cancer Inst.*, **54**, 157.

BLOCH-SHTACHER, N. & SACHS, L. (1976). Chromosome balance and the control of malignancy. *J. Cell Physiol.*, **87**, 89.

BLOCH-SHTACHER, N. & SACHS, L. (1977). Identification of a chromosome that controls malignancy in Chinese hamster cells. *J. Cell Physiol.*, **93**, 205.

CHEN, T.R. (1977). *In situ* detection of mycoplasma contamination in cell cultures by fluorescent Hoechst 33258 stain. *Exp. Cell Res.*, **104**, 255.

CODISH, S.D. & PAUL, B. (1974). Reversible appearance of a specific chromosome which suppresses malignancy. *Nature*, **252**, 610.

CONNELL, J.R. & OCKEY, C.H. (1977). Analysis of karyotype variation following carcinogen treatment of Chinese hamster primary cell lines. *Int. J. Cancer*, **20**, 768.

COWELL, J.C. (1980). A new chromosome region possibly derived from double minutes in an *in vitro* transformed epithelial cell line. *Cytogenet. Cell Genet.*, **27**, 2.

FOULDS, L. (1969). *Neoplastic Development*, **1**, Academic Press, London.

FOULDS, L. (1975). *Neoplastic Development*, **2**, Academic Press, London.

HARNDEN, D.G. (1977). Cytogenetics of human neoplasia. In *Genetics of Human Cancer* p. 87, (Eds. Mulvihill *et al.*) Raven Press, New York.

HITOTSUMACHI, S., RABINOWITZ, Z. & SACHS, L. (1971). Chromosomal control of reversion in transformed cells. *Nature*, **231**, 511.

HITOTSUMACHI, S., RABINOWITZ, Z. & SACHS, L. (1972). Chromosomal control of chemical carcinogenesis. *Int. J. Cancer*, **9**, 305.

KATO, R. (1968). The chromosomes of forty-two primary Rous sarcomas of the Chinese hamster, *Hereditas*, **59**, 63.

KATO, H. & YOSHIDA, T.H. (1972). Banding patterns of Chinese hamster chromosomes revealed by new techniques. *Chromosoma*, **36**, 272.

KIRKLAND, D.J. & VENITT, S. (1976). Chemical transformation of Chinese hamster cells. II. Appearance of market chromosomes in transformed cells. *Br. J. Cancer*, **34**, 145.

KLEIN, G. (1979). The role of viral transformation and cytogenetic changes in viral oncogenesis. In *Ciba Foundation Symposium* **66**, *Human Genetics: Possibilities and Realities*, p. 335, Excerpta Medica, Amsterdam.

LEHMAN, J.M. & TREVOR, K. (1979). Karyology and tumourigenicity of a Simian virus 40-transformed Chinese hamster cell clone. *J. Cell Physiol.* **98**, 443.

LEVAN, G. & LEVAN, A. (1975). Specific chromosome changes in malignancy: studies in rat sarcomas induced by two polycyclic hydrocarbons. *Hereditas*, **79**, 161.

LEVAN, G., MANDAHL, N., BENGISSON, B.O. & LEVAN, A. (1977). Experimental elimination and recovery of double minute chromosomes in malignant cell populations. *Hereditas*, **86**, 75.

LEVAN, A., LEVAN, G. & MANDAHL, N. (1978). A new chromosome type replacing the double minutes in a mouse tumour. *Cytogenet. Cell Genet.*, **20**, 12.

LEVAN, A. & LEVAN, G. (1980). Large double minutes with ring shape and rod shape. *Hereditas*, **92**, 259.

MACPHERSON, I.A. & MONTAGNIER, L. (1965). Agar suspension culture for the selective assay of cells transformed by polyoma virus. *Virology*, **23**, 291.

MARK, J. (1967). Double minutes – A chromosomal aberration in Rous sarcomas in mice. *Hereditas*, **57**, 1.

MITELMAN (1974). The Rouse sarcoma story: Cytogenetics of tumours induced by RSV In: *Chromosomes and Cancer* p. 675 (Ed. German) John Wiley & Son, New York.

NEWBOLD, R.F., OVERELL, R.W. & CONNELL, J.R. (1982). Immortality is an early event in the malignant transformation of mammalian cells by carcinogens. *Nature*, **299**, 633.

NOWELL, P.C. (1974). Chromosome changes and the clonal evolution of cancer. In *Chromosomes and Cancer*, p. 267, (Ed. German) John Wiley & Sons, New York.

ROWLEY, J.D. (1980). Chromosome abnormalities in cancer. *Cancer Genet. Cytogenet.*, **2**, 175.

SPRIGGS, A.I. (1974). Cytogenetics of cancer and precancerous states of the cervix uteri. In *Chromosomes and Cancer*, p. 423, (Ed. German) John Wiley & Sons, New York.

SUMNER, A.T., EVANS, H.J. & BUCKLAND, R.A. (1971). A new technique for distinguishing between human chromosomes. *Nature* (*New Biol.)*, **232**, 31.

TREWYN, R.W., KERR, S.J. & LEHMAN, J.M. (1979). Karyotype and tumorigenicity of 1-methylguanine-transformed Chinese hamster cells. *J. Natl Cancer Inst.*, **62**, 633.

YAMAMOTO, T., HAYASHI, M., RABINOWITZ, Z. & SACHS, L. (1973a). Chromosomal control of malignancy in tumours from cells transformed by polyoma virus. *Int. J. Cancer*, **11**, 555.

YAMAMOTO, R., RABINOWITZ, Z. & SACHS, L. (1973b). Identification of the chromosomes that control malignancy. *Nature* (*New Biol.*), **243**, 247.